# Using mobile phone technology to treat alcohol use disorder: study protocol for a randomized controlled trial

**DOI:** 10.1186/s13063-018-3137-y

**Published:** 2018-12-29

**Authors:** Anna-Karin Danielsson, Andreas Lundin, Sven Andréasson

**Affiliations:** 1 Department of Public Health Sciences, Karolinska Institutet, SE-171 77 Stockholm, Sweden; 2 Center for Psychiatric Research, Stockholm, Sweden

**Keywords:** Alcohol dependence, Randomized controlled trial, Mobile phone, App, Breathalyzer

## Abstract

**Background:**

A primary concern within the healthcare system is to make treatment more accessible as well as attractive for the great majority of alcohol-dependent people who feel reluctant to participate in the treatment programs available. This paper presents the protocol for a randomized controlled trial (RCT) to test the efficacy of two different technical devices (mobile phone application and breathalyzer) on alcohol consumption.

**Methods:**

The study is a three-armed RCT with follow-ups 3 and 6 months after randomization. In total, 375 adults (age 18+ years) diagnosed with alcohol use disorder (AUD) will be invited to participate in a 3-month intervention. The primary outcome is the number of days with heavy drinking, defined as four or more standard drinks (12 g alcohol/drink) and measured by the timeline follow back (TLFB) and Alcohol Use Disorder Identification Test (AUDIT) instruments at 3-month and 6-month follow-up. Secondary outcome measures include weekly alcohol consumption, measured by the TLFB, AUDIT, and phosphatidylethanol in blood values at 3-month and 6-month follow-up (number of days with blood alcohol concentration levels exceeding 60 mg/100 ml).

**Discussion:**

Improving ways of collecting data on alcohol consumption, as well as the treatment system with regards to AUD, is of vital importance. Mobile phone technology, with associated applications, is widely recognized as a potentially powerful tool in the prevention and management of disease. This study will provide unique knowledge regarding the use of new technology as instruments for measuring alcohol consumption and, also, as a possible way to decrease it.

**Trial registration:**

ISRCTN, ISRCTN14515753. Registered on 31 May 2018.

**Electronic supplementary material:**

The online version of this article (10.1186/s13063-018-3137-y) contains supplementary material, which is available to authorized users.

## Background

It is well known that alcohol causes significant morbidity and mortality [1]. Approximately 70 diseases have been estimated to be wholly or partly caused by alcohol [2]; for example, cardiovascular diseases, cancers, infectious diseases, neurological diseases, and mental disorders—including alcohol use disorder (AUD). AUD is a disease where at least three out of six ICD-10 criteria should have occurred together for at least 1 month or, if persisting for periods of less than 1 month, should have occurred together repeatedly within a 12-month period. The criteria include a strong *desire* to drink alcohol, *impaired* capacity to *control* alcohol-taking, *preoccupation* with alcohol use, *persistent* substance use *despite* clear evidence of *harmful* consequences, a physiological *withdrawal state*, and evidence of *tolerance*.

Most people with alcohol use disorder do not seek treatment [3, 4]. Different studies estimate that fewer than 20% have ever been in treatment [5, 6]. Available treatment in specialized addiction clinics is perceived as unattractive and stigmatizing, and it appears that it is only when problems become very severe that the barriers to treatment are overcome [7].

Thus, a primary concern within the healthcare system is to make treatment more accessible as well as attractive for the great majority of alcohol-dependent people who feel reluctant to participate in the traditional treatment programs available.

In recent years, a number of technical devices have been developed for this particular purpose; for example, web-based preventative and treatment self-help programs aiming at reducing alcohol use and/or treating AUD [8]. Internet-based interventions are typically well received by clients and may attract individuals who would otherwise not seek help, but prior research on their effectiveness is inconsistent [9]. Moreover, and even more recent, mobile phone technology is getting widely recognized as a potentially powerful tool for the prevention and management of disease [10]. Increased accessibility, real-time and ecological assessments, as well as high allowance for collecting sensitive information are some of the advantages of smartphone applications. There is, however, little research to provide evidence of their putative effectiveness [11, 12].

So far, mobile technology and health-related apps have been evaluated with regards to diabetes, infectious disease (HIV, tuberculosis), dermatology (psoriasis), and smoking [13–17]. Studies on the topic have also been made within the field of mental health, including depression, sleep disturbances, anxiety, and self-harm [18]. To our knowledge, there are only two previously published studies regarding tests of apps in the treatment of alcohol use disorder; the Location-Based Monitoring and Intervention for Alcohol Use Disorders (LBMI-A) and the Addiction—Comprehensive Health Enhancement Support System (A-CHESS). The LBMI-A app has features intended to provide support, and in a study population of 28 individuals with an alcohol use disorder the tool for monitoring consumption was appreciated [19]. For the other app, A-CHESS, results from a randomized controlled trial of 349 individuals showed that patients using the app (*n* = 170) reported less risky drinking than the controls (*n* = 179) [20]. The main criticism regarding previous research in this area concerns sample size, high risk of bias, and lack of studies considering long-term follow-up [12, 18].

In this project we aim to examine two mobile phone applications (apps), “Glasklart” and the combination of a portable breathalyzer with a mobile phone application “iBAC”, in the treatment of AUD. The Glasklart app enables the user to make instant, continuous, and anonymous registration of the alcohol volume (i.e. standard drinks) consumed on each occasion, with the possibility to also share information with a caregiver. The advantages of using a smartphone app are several; smartphones may store and share data in real time, give geographical location, allow for on-demand communication, are almost always on, and are portable [21]. The iBAC device gives a biologic measure of the current blood alcohol concentration (BAC). The advantage is that this provides an estimate that is independent of self-report and that this device is also easily managed using a mobile phone. In general, alcohol consumption data are collected in retrospect, thus increasing the risk of recall bias [22]. Possible consequences of incorrect information regarding alcohol consumption are several; for example, difficulties in clinically assessing alcohol-related problems and evaluating treatment of alcohol-related problems for the individual, and difficulties in examining the health effects of alcohol consumption. This project enables *instant* assessment of alcohol consumption which in turn may allow for more accurate reports.

We will conduct a randomized controlled trial (RCT) to examine the effects of using apps as complements to standard treatment on alcohol consumption in adults with AUD. A comparison group will receive standard treatment only, described later. Specifically, we aim to validate Glasklart and iBAC as instruments for assessing alcohol consumption, and to investigate whether assessment (self-monitoring), using Glasklart or iBAC, has a reducing effect on alcohol consumption.

### Key research questions

1. Do mobile phone applications provide better (i.e., higher) estimates of alcohol consumption than current measurement methods?
2. Does the use of these devices, on the one hand featuring self-monitoring of alcohol consumption, and on the other measuring blood alcohol levels through a breathalyzer connected to a mobile phone, have an effect on the user’s alcohol consumption?
3. How are Glasklart and iBAC perceived by the users? This question includes assessments of the technical components, such as app features including reminders, geolocalization, and registration of mood (qualitative study).

## Methods

**Fig. 1:**
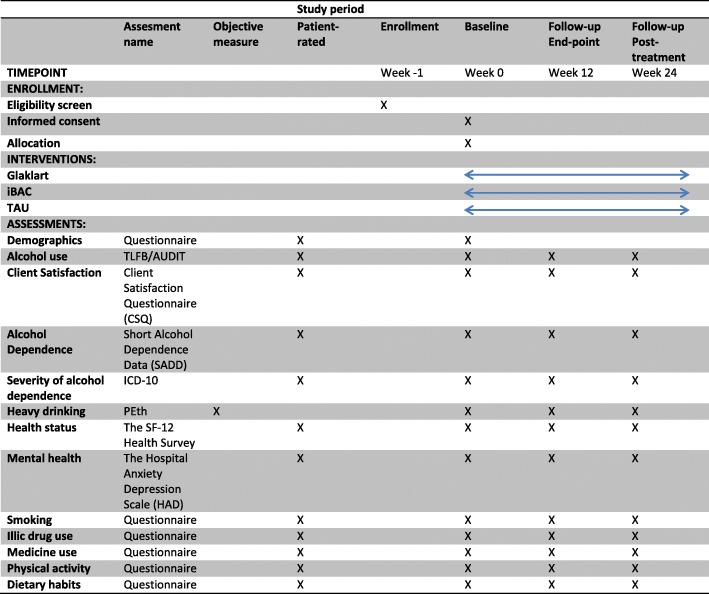
Standard Protocol Items: Recommendations for Interventional Trials (SPIRIT) figure. AUDIT Alcohol Use Disorder Identification Test, ICD-10 International Statistical Classification of Diseases and Related Health Problems 10th revision, PEth phosphatidylethanol, SF-12 12-item Short form Health Survey, TAU treatment as usual, TLFB timeline follow back

This study will be conducted and reported in accordance with the Standard Protocol Items: Recommendations for Interventional Trials (SPIRIT) guidelines (see Fig. 1 and Additional file 1 for the SPIRIT figure and checklist, respectively).

### Setting and participants

The study will be conducted at Riddargatan 1: Center for Alcohol and Health, an outpatient treatment clinic located in central Stockholm specializing in AUDs. The clinic opened in 2011 and is staffed by physicians, psychologists, and allied health workers with expertise in the treatment and management of addictive behaviors, including AUDs. The target group at Riddargatan 1 is people with alcohol dependence but no major psychosocial problems.

#### Inclusion/exclusion

Inclusion criteria were fulfilling diagnostic criteria for alcohol dependence according to ICD-10 and age 18 years or older.

Exclusion criteria were severe physical or mental disorder, pregnancy, currently undergoing other treatment for alcohol problems, and recent treatment for severe alcohol problems (e.g., alcohol withdrawal).

### Study design and randomization

The study comprises three parts:

1. A randomized controlled trial, measuring the effect on alcohol consumption of adding the two devices Glasklart and iBAC to treatment as usual (TAU) at Riddargatan 1.
2. A validation study of Glasklart and iBAC.
3. A qualitative study, where focus groups and semi-structured interviews will be used to study participants’ perceptions of these technological tools.

#### Randomization

**Fig. 2:**
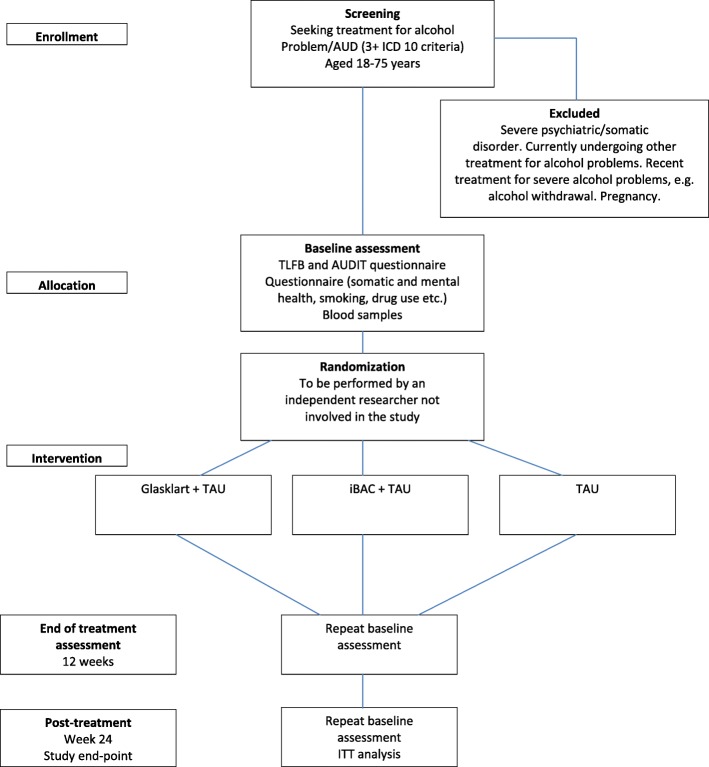
Participant flow diagram. AUD alcohol use disorder, AUDIT Alcohol Use Disorder Identification Test, ICD 10 International Statistical Classification of Diseases and Related Health Problems 10th revision, ITT intention to treat, TAU treatment as usual, TLFB timeline follow back

Following the first treatment session, the counselor informs the study coordinator about the new study participant (see Fig 2 for participation flow diagram). The coordinator records baseline data in a study database and initiates the randomization procedure. This procedure is conducted by an administrator with no other role in this study. Randomization is done by a computer program, where participants are randomized (in blocks of 10) either to treatment as usual (TAU), or TAU + Glasklart, or TAU + iBAC. TAU in this study at Riddargatan 1 involves pharmacotherapy in combination with manualized psychological treatment—either the “Guide to better drinking habits”, building on theories on guided self-change [23–25], or the “Guide to Controlled drinking”, referring to theories on behavioral self-control training [26]. Each study participant is given a study number (1–375). Only the administrator has access to the code key, where the study number is coupled with the participant’s person number. The code key is stored in a locked cabinet in the patient archive at the clinic.

At the second treatment session, the counselor collects the signed informed consent document. The counselors then explain and demonstrate the two devices: Glasklart and iBAC. Each patient randomized to the iBAC intervention is provided with an iBAC breathalyzer (one of the smallest breathalyzers available) for the duration of the study. The apps (Glasklart and iBAC respectively) will be installed on the participants’ smartphones. The iBAC breathalyzer is linked to the mobile phone via Bluetooth and the information on the BAC level is saved in the breathalyzer and mobile app. The user identifies herself or himself using a color-coding photographic function to verify that the intended user takes the test.

Patients randomized to Glasklart will be instructed to register every glass of alcohol they drink in the Glasklart app. They will receive automatic reminders, a so-called push notification that pops up on the mobile device after half an hour (after first registration), to continue registration if they have consumed more alcohol (i.e., “do you have anything more to register?”). Patients randomized to iBAC treatment will also receive a message on their mobile phone instructing them to use their iBAC breathalyzer at hourly intervals during drinking occasions, as long as these go on. That is, the iBAC is programmed to contact the patient with push notification according to a preset time schedule. The patients can then review their drinking through the apps respectively. Glasklart will describe the number of drinks consumed, when and where, in what mood, and whether alone or together with others. iBAC will describe the BAC level at different time points. Both of these devices can be viewed on a daily, weekly, or monthly basis. Moreover, the information collected is also visible for the counselors.

At all sessions of the treatment program, at baseline, at 3 months, and at a follow-up after 6 months, the timeline follow back (TLFB) and AUDIT will be recorded, and blood tests will be analyzed for phosphatidylethanol in blood (PEth).

#### Blinding

A team of psychiatric nurses at the clinic will be trained to conduct patient assessments before, during, and after treatment. Assessors will be aware of the patient’s treatment allocation. Genuine blinding of assessors is not feasible given the technical devices that need to be displayed in the project. Thus, blinding will not be possible at the patient or therapist level. At the analysis stage, however, the participants’ identity will not be known to the researcher; that is, all data will be available according to group allocation (1, 2, or 3).

### Procedure

Recruitment will be done at Riddargatan 1, where people starting a treatment program for alcohol dependence will be informed about the study by the physician in charge. Patients expressing willingness to participate will be included. Information about the devices under study and the study procedures is provided by the counselor in charge of the treatment at the first treatment session, after assessing inclusion and exclusion criteria. Recruitment will also occur through advertisements placed in the waiting room.

To improve adherence, data on participant use of the devices will be continuously monitored. If necessary, the study coordinator will send reminders via text messages to the participants. Also, the devices will generate reminders (e.g., “push notifications”) for the participants.

When participants are included in the study they will be given timings for follow-up meetings at 3 and 6 months after study baseline. The follow-up visits will be with the study coordinator. All study questionnaires will be stored in a Case Record Form (CRF). Between the study visits, all CRFs will be kept in a locked and secure data archive room.

The counselors involved in this study are all certified therapists, with relevant specialist training for the treatment programs chosen for this study; that is, pharmacological treatment combined with the “Guide to better drinking habits” or the “Guide to Controlled drinking”, both based on cognitive behavioral treatment. Medications that will be used in this study are naltrexone and acamprosate.

The project is planned to start in 2018. Participant recruitment is expected to be completed by the end of 2019. Follow-ups will be completed by July 2020. Results will be reported during the fall of 2020.

### Baseline and follow-up assessments

Baseline data for this study, the TLFB interview, the AUDIT questionnaire, and the biomarker PEth, as well as diagnostic instruments for the diagnosis of alcohol dependence are collected routinely at intake for all patients at Riddargatan 1.

#### RCT outcome measures

##### Primary outcome measure

The primary outcome is the number of days with heavy drinking, defined as four or more standard drinks (12 g alcohol/drink), measured by the timeline follow back instrument (TLFB) and AUDIT.

##### Secondary outcome measures

The secondary outcomes are weekly alcohol consumption, measured by TLFB and AUDIT, and PEth values in blood at 3-month and 6-month follow-up, and the number of days with BAC levels exceeding 60 mg/100 ml.

#### Validation study

The validation study will correlate registered consumption in the Glasklart app and iBAC respectively, with results from the TLFB, AUDIT, and PEth. Agreement will also be tested using the intra-class correlation (ICC) and kappa coefficient statistics, for consumption structured as groups.

#### Qualitative study

The qualitative study will include focus group interviews and individual interviews focusing on study participants’ perceptions of these technological tools (e.g., Are Glasklart or iBAC easy to use? Are they perceived as good support/help in the treatment?).

#### Instruments

- Demographics: age, gender, occupation, and marital status (baseline only).
- Timeline follow back: structured interview for assessment of alcohol consumption during the last 30 days [27].
- Alcohol Use Disorder Identification Test (AUDIT) questionnaire [28], modified to the 3-month reference period in the follow-up.
- Severity of alcohol dependence is measured by the number of fulfilled diagnostic criteria for the diagnosis alcohol dependence, according to ICD-10.
- Short Alcohol Dependence Data (SADD) [29, 30]: this instrument is currently undergoing Swedish validation and will be published during 2018.
- Client Satisfaction Questionnaire (CSQ) [31].
- The Hospital Anxiety Depression Scale (HAD) [32]: 14 items scored 0–3 based on how the respondent felt during the past week.
- The Short Form Health Survey (SF-12) [33]: 12 items assess functional health and wellbeing from the respondent’s perspective.
- Phosphatidylethanol (PEth): blood samples will be collected by staff at Karolinska Universitetssjukhusets Laboratorium (KS Lab) in Stockholm and will be analyzed at KemLab/LS Laboratorium. The results will be delivered through the electronic patient record system (Take Care) within 2–3 days. The results will be printed and anonymized by the study coordinator and then placed in the CRF.

### Statistical analyses and power calculation

The sample size has been estimated from the primary outcome variable, heavy drinking days during the past 30 days before baseline, at 3 months and at 6 months after baseline. Based on the assumption that the effect size of the intervention will be in the range of 0.4 SD in groupwise comparisons (alpha 5% and two-tailed tests) we need at least 98 individuals in each of the three treatment arms, and given an expected 25% dropout rate this motivates our choice of *N* = 375.

Data will be analyzed according to intention to treat (ITT). A secondary analysis will be per protocol for those participants who have contributed data at baseline and at 3-month follow-up. Primary and secondary outcome measures will be analyzed through ANOVA for repeated measures, with time (baseline, 3 months, and 6 months) as the dependent variable and type of intervention (TAU, or TAU + Glasklart, or TAU+ iBAC) as the independent variable.

A multiple regression analysis will be performed to investigate the extent to which the different predictor variables (consumption level and pattern, severity of dependence) contribute to the variation in the primary and secondary outcome measures.

## Discussion

The high user degree of mobile phones, close to a 96% penetration rate worldwide, suggests a huge potential to reach populations with mobile technology [34]. In 2012 alone, a download of 40,000 different health-related apps was reported [21], a number most likely to be on a continuous rise. Thus, mobile phone technology is widely recognized as a potentially powerful tool for the prevention and management of disease [10]. Increased accessibility, real-time and ecological assessments, as well as high allowance for collecting sensitive information are some of the advantages of smartphone applications. There is, however, little research to provide evidence of its putative effectiveness [11, 12]. Few studies have been carried out, and criticism has been brought forth regarding small sample sizes, not considering possible biases and/or short follow-up times [18].

Improving ways of collecting data on alcohol consumption, as well as the treatment system, is of vital importance for the clinical practitioner as well as for the public health specialist. Today, there are uncertainties regarding reported levels of alcohol consumption for both total alcohol intake and pattern of drinking, and consequently also in assessing health effects of alcohol and in evaluating possible effects of treatment [22, 35]. Using digital technology has been shown to be effective not only in decreasing barriers for individuals to seek treatment but also with regards to the outcome of treatment, where it has been shown to be as effective as face-to-face alternatives [36]. Mobile phone technology has also been suggested as one means to offer time-efficient support within health care [37]. In conclusion, making use of novel technology within health care could be beneficial not only for the individual but also for society at large.

### Ethical considerations

Using apps which target the consumption of alcohol might be negative for some. One previous study suggests that male participants using the app “Promille-kollen” increased their drinking frequency and the authors speculated that use of a smartphone app might trigger men to compete with their peers in a competitive “drinking game” [38]. However, this study was in a university setting, enabling comparison between participants, while our study is in a clinical setting with individual participation only. Both apps used within our research project are developed by Med-tech companies specializing in e-health/m-health solutions for health organizations and other companies. Although in practice it would not only be possible but also very easy to make these apps publicly available for everyone to use (via App store or Google play), this will hardly be the case considering these developmental companies aim to make a profit. A possible future scenario is that m-health devices like these are made available via licenses that are procured by the healthcare systems. Currently, there are no ethical guidelines for mobile health applications (apps) despite the rapid innovation and use in the healthcare field. With regards to the suggested project, all collected data will be managed in a manner that is compatible with the security and personal data law. No personal data will be stored by Glasklart or iBAC, only a study number. Participation in the suggested study is considered not to present any risks for the patients; participation is voluntary and patients have sought treatment for their alcohol consumption.

This clinical trial will provide unique knowledge regarding the use of an application in a medical setting, by validating the app Glasklart and the breathalyzer iBAC as instruments for measuring alcohol consumption and, also, the possible effects that self-monitoring may have on the participants’ alcohol consumption behavior. The planned studies will also contribute information regarding how some possible app-specific features are perceived by the user, such as time-sensitive reminders, geolocation, and registering of mood and cravings when consuming alcohol. Possible implications for treatment of AUD will be discussed in future papers.

## Trial status

The trial is due to commence in 2018.

Protocol version 1, 31 May 2018.

Protocol amendments will be reported to the ISRCTN registry. http://www.isrctn.com/ISRCTN14515753

## Additional file

Additional file 1: SPIRIT 2013 checklist: recommended items to address in a clinical trial protocol and related documents (DOC 121 kb)

## Abbreviations

A-CHESS: Addiction—Comprehensive Health Enhancement Support System
AUD: Alcohol use disorder
BAC: Blood alcohol concentration
CRF: Case Record Form
CSQ: Client Satisfaction Questionnaire
HAD: Hospital Anxiety Depression Scale
ICC: Intra-class correlation
ITT: Intention to treat
LBMI-A: Location-Based Monitoring and Intervention for Alcohol Use Disorders
PEth: Phosphatidylethanol
RCT: Randomized controlled trial
SADD: Short Alcohol Dependence Data
SF-12: Short form Health Survey
TAU: Treatment as usual
TLFB: Timeline follow back
